# A Case Report and Literature Review on Dietary Practices and Megaloblastic Anemia in a Young Female: Unraveling the Impact of Nutrition on Hematological Health

**DOI:** 10.7759/cureus.61550

**Published:** 2024-06-02

**Authors:** Devshree Dhande, Archana Dhok, Ashish Anjankar, Shailesh Nagpure

**Affiliations:** 1 Biochemistry, Jawaharlal Nehru Medical College, Datta Meghe Institute of Higher Education and Research, Wardha, IND; 2 Pharmacology, Jawaharlal Nehru Medical College, Datta Meghe Institute of Higher Education and Research, Wardha, IND

**Keywords:** dietary practices, hematological health, veganism, vegetarianism, vitamin b12 deficiency, megaloblastic anemia

## Abstract

Megaloblastic anemia, stemming from vitamin B12 or folate deficiencies, poses diagnostic challenges due to its diverse clinical presentation. We report a case of a 25-year-old female college student presenting with symptoms indicative of megaloblastic anemia, attributed to her recent adoption of a strict vegetarian and vegan diet. Clinical manifestations included dizziness, palpitations, blurred vision, vertigo, headaches, burning sensations, excessive sweating, mouth ulcers, and unintentional weight loss. Physical examination revealed pale palpebral conjunctiva and sweating on the palms and soles. Laboratory findings confirmed megaloblastic anemia secondary to vitamin B12 deficiency, with elevated mean corpuscular volume (MCV), reticulocyte count, serum methylmalonic acid (MMA), and homocysteine levels. Treatment with intramuscular cyanocobalamin injections and oral vitamin B12 supplementation led to symptomatic improvement and normalization of hematological parameters. This case underscores the crucial role of dietary habits in hematological health. Vegetarian and vegan diets, devoid of animal products rich in vitamin B12, increase the risk of deficiency. Early recognition and management of such deficiencies are imperative to prevent long-term complications. A literature review corroborates the association between vegetarianism/veganism and megaloblastic anemia risk. Healthcare providers should vigilantly assess dietary histories, particularly in patients with hematological abnormalities. Further research is warranted to explore strategies for optimizing nutrient intake in individuals adhering to vegetarian or vegan diets, aiming to mitigate the risk of nutritional deficiencies and associated complications.

## Introduction

Megaloblastic anemia is a hematologic disorder characterized by large, immature, and dysfunctional red blood cells in the bone marrow and peripheral blood [1]. This condition primarily arises from impaired DNA synthesis and subsequent ineffective erythropoiesis, resulting in macrocytosis and anemia. The most common etiologies of megaloblastic anemia are deficiencies in vitamin B12 (cobalamin) and folate (vitamin B9), essential micronutrients involved in nucleic acid synthesis and cellular proliferation [1,2]. Vitamin B12 plays a critical role in numerous physiological processes, including the metabolism of amino acids and fatty acids and the maintenance of the nervous system [3]. Dietary sources of vitamin B12 are predominantly derived from animal products such as meat, poultry, fish, eggs, and dairy. As such, individuals adhering to vegetarian or vegan diets, which exclude or limit animal-derived foods, are at an increased risk of developing vitamin B12 deficiency [4].

Conversely, folate is abundant in green leafy vegetables, legumes, fruits, and fortified grains [5]. However, inadequate dietary intake, malabsorption, or increased demand (e.g., during pregnancy or in cases of rapid cell turnover) can lead to folate deficiency, contributing to the development of megaloblastic anemia [6]. The clinical presentation of megaloblastic anemia can vary widely, ranging from asymptomatic cases detected incidentally on routine blood tests to severe cases manifesting with symptoms such as fatigue, weakness, pallor, dyspnea, palpitations, glossitis, neurologic deficits, and gastrointestinal disturbances [7]. Prompt recognition and treatment of megaloblastic anemia are crucial to prevent complications and improve patient outcomes. Several studies have investigated the association between dietary practices, particularly vegetarianism and veganism, and the risk of megaloblastic anemia [8]. These investigations have highlighted the importance of adequate vitamin B12 and folate intake in maintaining hematological health, especially in populations with dietary restrictions. Additionally, research has focused on identifying effective strategies for screening, diagnosis, and management of megaloblastic anemia, including the role of oral and parenteral vitamin supplementation [9-12].

In this context, we present a case report and literature review that underscores the impact of dietary practices on hematological health, focusing on the development of megaloblastic anemia in a young female following a strict vegetarian and vegan diet. Through an exploration of the clinical presentation, diagnostic evaluation, therapeutic interventions, and outcomes of this case, as well as a comprehensive review of the existing literature, we aim to elucidate the complex interplay between nutrition and hematopoiesis, emphasizing the importance of early recognition and management of nutritional deficiencies to optimize patient care.

## Case presentation

A 25-year-old female, a college student, presented with a constellation of symptoms over the past month. She complained of dizziness, palpitations, blurred vision, vertigo, headaches, burning sensations, and excessive sweating from the palms and soles. Additionally, she reported frequent occurrences of mouth ulcers and notable tiredness, accompanied by an unintentional weight loss of approximately 2 kg within the same timeframe. Notably, the patient had recently attended a meditation center for three months, during which she adhered strictly to a vegetarian and vegan diet, abstaining from non-vegetarian foods and dairy products despite being a non-vegetarian otherwise. There was neither a significant past medical history nor any family history of note.

**Figure 1:**
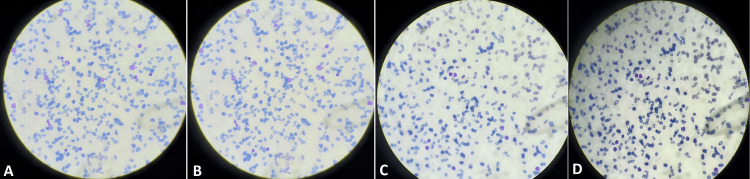
(A) Macrocytic RBCs, (B) hypersegmented neutrophil, (C) anisocytosis, (D) poikilocytosis RBCs: red blood cells

Upon physical examination, the patient exhibited sweating on her palms and soles and a slightly pale palpebral conjunctiva. Vital signs were within normal limits, with a blood pressure reading of 111/76 mmHg and a respiratory rate of 15 breaths per minute. A mental status examination revealed the patient to be oriented to time, place, and person, with no remarkable findings on the systemic examination. Laboratory investigations unveiled significant abnormalities in her hematological parameters. Her hemoglobin and hematocrit levels were markedly decreased, measuring 8 mg/dL and 17%, respectively, indicative of anemia. The mean corpuscular volume (MCV) was elevated at 93 fL, suggesting macrocytic red blood cells. The reticulocyte count was elevated, as was the red blood cell distribution width (RDW), indicating anisocytosis. Additionally, thrombocytopenia was noted, with a platelet count of 149,000/mL. Biochemical investigations revealed a decreased serum vitamin B12 level of 190 pg/mL, along with elevated levels of serum methylmalonic acid (MMA) and homocysteine, further supporting the diagnosis of vitamin B12 deficiency. Histopathological examination depicted macrocytic red blood cells (RBCs), hypersegmented neutrophils, anisocytosis, and poikilocytosis (Figure 1).

Based on the clinical presentation and laboratory findings, a diagnosis of megaloblastic anemia secondary to vitamin B12 deficiency was made. Other potential causes of megaloblastic anemia, such as folate deficiency, alcoholism, liver disease, and medications affecting DNA synthesis, were ruled out through comprehensive patient history, clinical examination, and specific laboratory tests. The patient was initiated on therapeutic intervention with intramuscular cyanocobalamin injections at a dose of 1,000 mcg once a week for four weeks, followed by 1,000 mcg monthly for four months. Additionally, oral vitamin B12 supplementation was prescribed at a dose of 1,000 mcg daily for six months. Following one month of treatment, the patient reported significant improvement in her symptoms, with resolution of dizziness, palpitations, blurred vision, vertigo, headaches, burning sensations, excessive sweating, and mouth ulcers. Hemoglobin and hematocrit levels showed marked improvement. At the five-month follow-up, the patient remained asymptomatic, with normalization of her vitamin B12 levels, and no significant complaints were reported. This case underscores the importance of considering dietary habits, such as strict vegetarian or vegan diets, in evaluating anemia and other nutritional deficiencies. Early recognition and prompt treatment of vitamin B12 deficiency can lead to rapid resolution of symptoms and improvement in overall patient outcomes.

## Discussion

Megaloblastic anemia, characterized by macrocytic RBCs, is often attributed to vitamin B12 or folate deficiencies, crucial nutrients involved in DNA synthesis and erythropoiesis. This discussion delves into the implications of the presented case and the broader literature regarding the impact of dietary practices, particularly vegetarianism and veganism, on hematological health. The case of the 25-year-old female underscores the intricate interplay between dietary choices and hematological manifestations. Her adoption of a strict vegetarian and vegan diet devoid of animal products led to vitamin B12 deficiency, resulting in megaloblastic anemia. This observation aligns with numerous studies demonstrating an increased risk of nutritional deficiencies, including vitamin B12 deficiency, among individuals adhering to plant-based diets [4,13-15]. Moreover, the severity of the patient's symptoms highlights the importance of early recognition and intervention to prevent long-term complications associated with megaloblastic anemia [7,16].

The literature review elucidates the mechanisms underlying the association between vegetarianism/veganism and hematological abnormalities. A systematic review by Pawlak et al. found that vegetarians and vegans have significantly lower serum vitamin B12 levels compared to omnivores [17]. Similarly, studies by Gilsing et al. [18] and Rosell et al. [19] demonstrated a higher prevalence of vitamin B12 deficiency among vegetarians and vegans. These findings underscore the need for dietary strategies to mitigate the risk of nutritional deficiencies in plant-based diets, such as fortification or supplementation with vitamin B12 [20,21]. Furthermore, the discussion extends to the clinical implications of vitamin B12 deficiency and megaloblastic anemia. Beyond hematological manifestations, untreated vitamin B12 deficiency can lead to neurological complications, including peripheral neuropathy and cognitive impairment [22,23]. Hence, early diagnosis and intervention are imperative to prevent irreversible neurological sequelae. The efficacy of vitamin B12 supplementation in reversing hematological abnormalities and improving clinical symptoms, as observed in the presented case, corroborates existing evidence supporting the importance of timely intervention [24,25].

Moreover, the discussion addresses the broader public health implications of dietary patterns on hematological health. With the growing popularity of vegetarian and vegan diets worldwide [26], healthcare providers must be vigilant in assessing and addressing potential nutritional deficiencies in their patient populations. Nutritional education and counseling play a pivotal role in promoting balanced dietary habits and preventing micronutrient deficiencies [27]. Additionally, further research is warranted to explore innovative approaches, such as fortified plant-based foods or alternative sources of vitamin B12, to optimize nutrient intake in individuals adhering to vegetarian or vegan diets [28,29].

## Conclusions

In conclusion, this case highlights the critical role of dietary assessment in diagnosing and managing nutritional deficiencies. Early intervention, including appropriate supplementation, is essential in preventing the complications associated with vitamin B12 deficiency. This case exemplifies the need for a comprehensive approach to patient care, ensuring optimal hematological health and overall well-being.
